# Supplementary figures and images for: Structural dynamics of SARS-CoV-2 nucleocapsid protein induced by RNA binding
Source: PLoS Comput Biol. 2022 May 12;18(5):e1010121. doi: 10.1371/journal.pcbi.1010121 (PMC9129039; doi:10.1371/journal.pcbi.1010121)

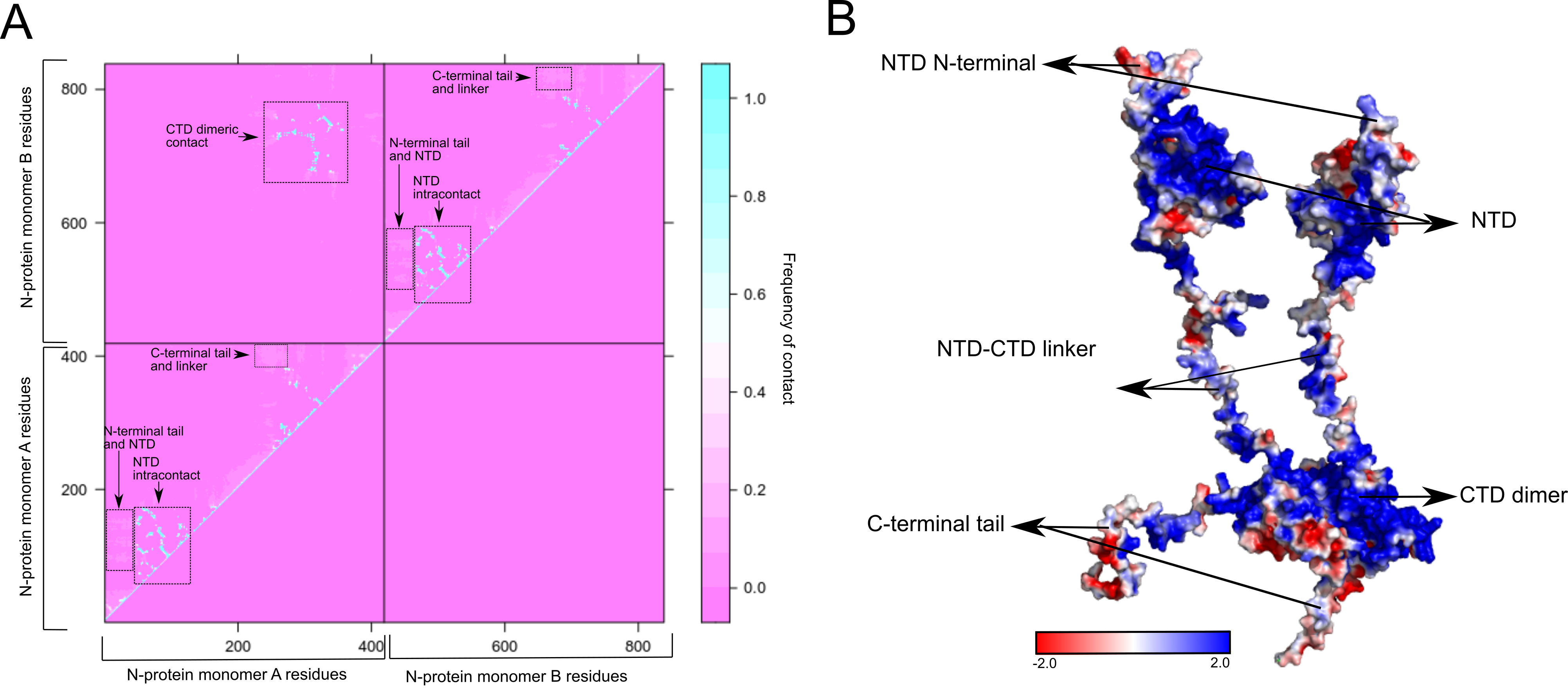

Supplement: S1 Fig — A- Contact map of the N protein dimer in RNA-free CG simulation. Contact map was built using Bio3D packaged. Five independent CG simulation runs were considered during frequency contact calculation and a cut-off distance between CG Cα beads was set to 10 Å. Relevant contact regions are indicated. B- Electrostatic potential surface of an extended conformation of the N protein dimer model. (TIF) [file pcbi.1010121.s001.tif]

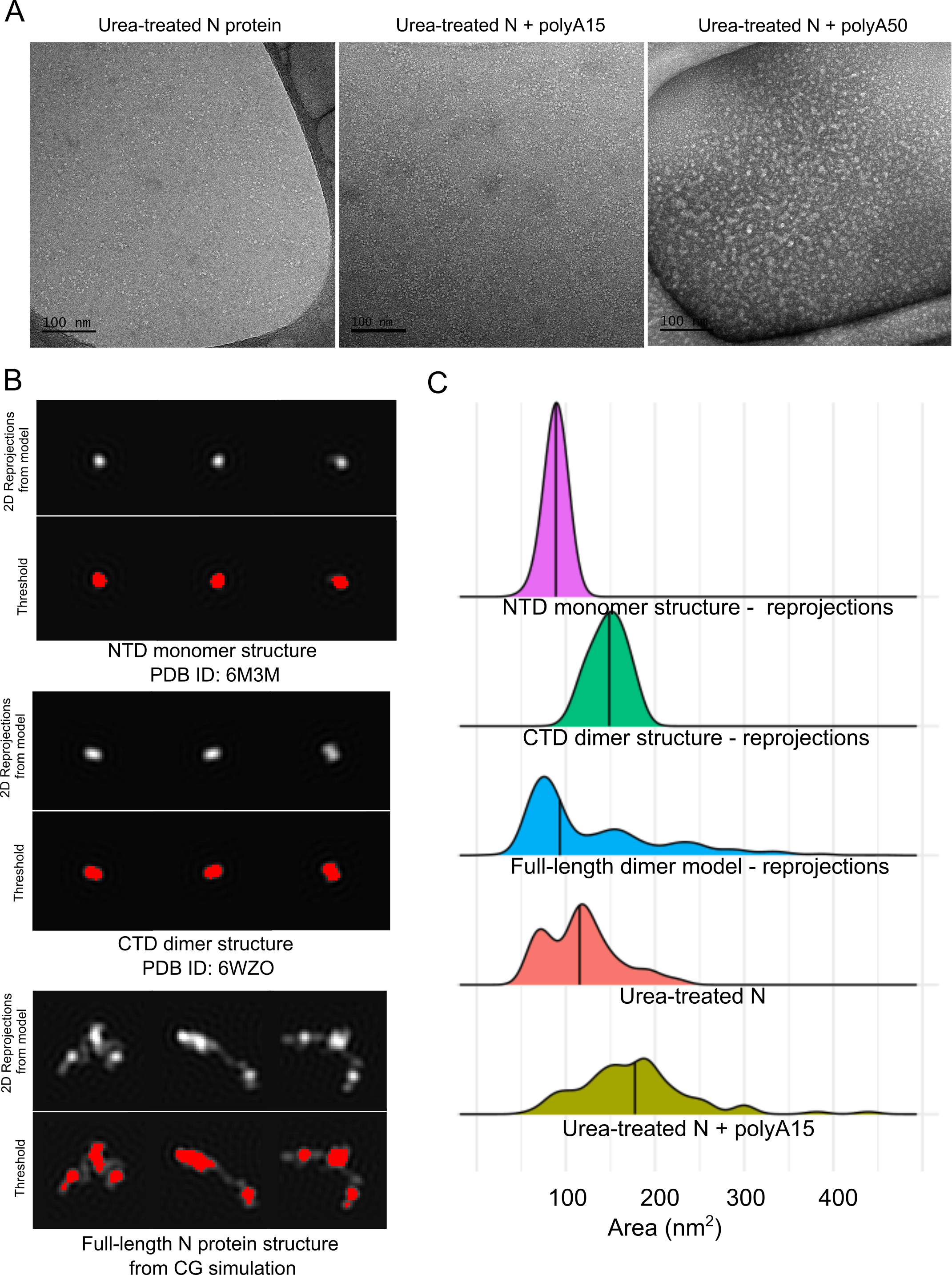

Supplement: S2 Fig — A- Representative micrographs for each condition: urea-treated N protein in absence of RNA (left panel), or in the presence of polyA15 (middle panel) or polyA50 (right panel) at a 1:10 protein-RNA ratio. Scale bars are indicated. B–Representative 2D simulated reprojections in three different orientations of NTD monomeric crystallographic structure obtained from PDB 6M3M (upper panel), CTD dimeric crystallographic structure obtained from PDB 6M3M (middle panel) and full-length N protein structured obtained from CG simulations (bottom panel). Below each reprojections are presented the threshold applied to identify the high-density regions in images, which corresponds to the structured domains of N protein. In full-length N protein reprojections, under the threshold used we did not include the flexible regions of N protein that has low intensity value. C–Area distribution of 3D structures (NTD monomer and CTD dimer) or 3D model reprojections (full-length dimer) and class averages of particles collected from negative stain images of urea-treated N samples, same as in Fig 2B. Here, the area distribution of urea-treated N with polyA15 samples, were included in the comparison presented in Fig 2B of the manuscript. The area distribution of urea-treated N overlaps the area distribution of NTD and CTD reprojections, indicating that the particles observed in the negative stain images corresponds to these individual domains. The presence of polyA15 shifted the area distribution of urea-treated N, indicating that the particles have larger dimensions than without RNA, but still compatible with the larger dimensions observed in full-length dimer reprojections. This finding suggests that in presence of polyA15, the individual NTD and CTD domains got closer and the individual density regions that are separated, got together and formed a unique particle with larger dimensions in comparison to the individual domains. A 2D classification of the N protein + polyA50 particles could not b [file pcbi.1010121.s002.tif]

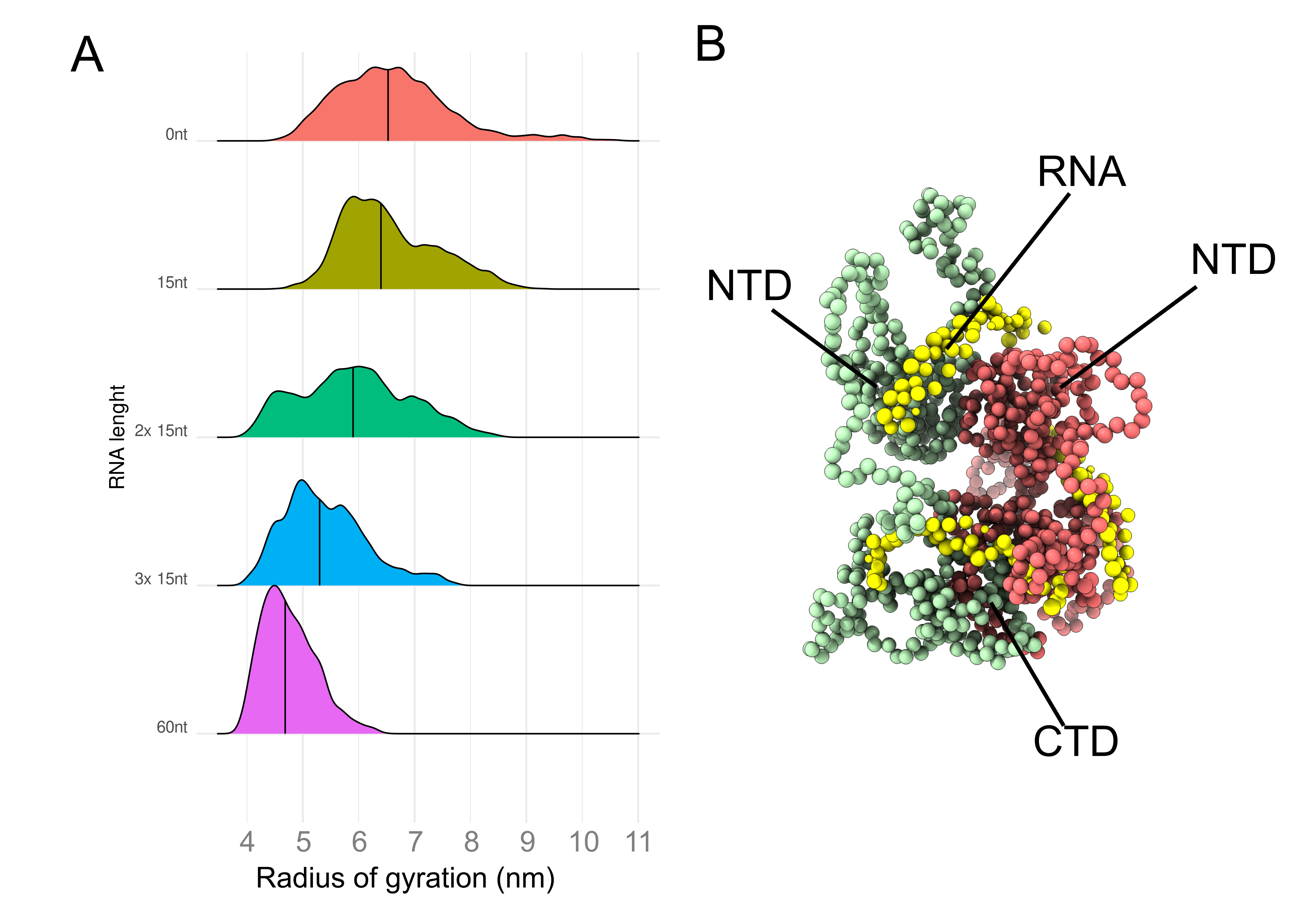

Supplement: S3 Fig — A- Density plot of the gyration radius distribution (in nm) calculated from five independent CG simulations of the N protein dimers in the presence of one, two or three single-strands 15-nt RNA. For comparison, we also presented distributions of conditions without RNA (0 nt) and in the presence of 60 nt long. B- Representative frame with minimum radius of gyration of full-length N dimer in complex with three 15-nt RNA molecules. N protein monomers are colored in red and green, whereas the RNA molecules are colored in yellow. (TIF) [file pcbi.1010121.s003.tif]

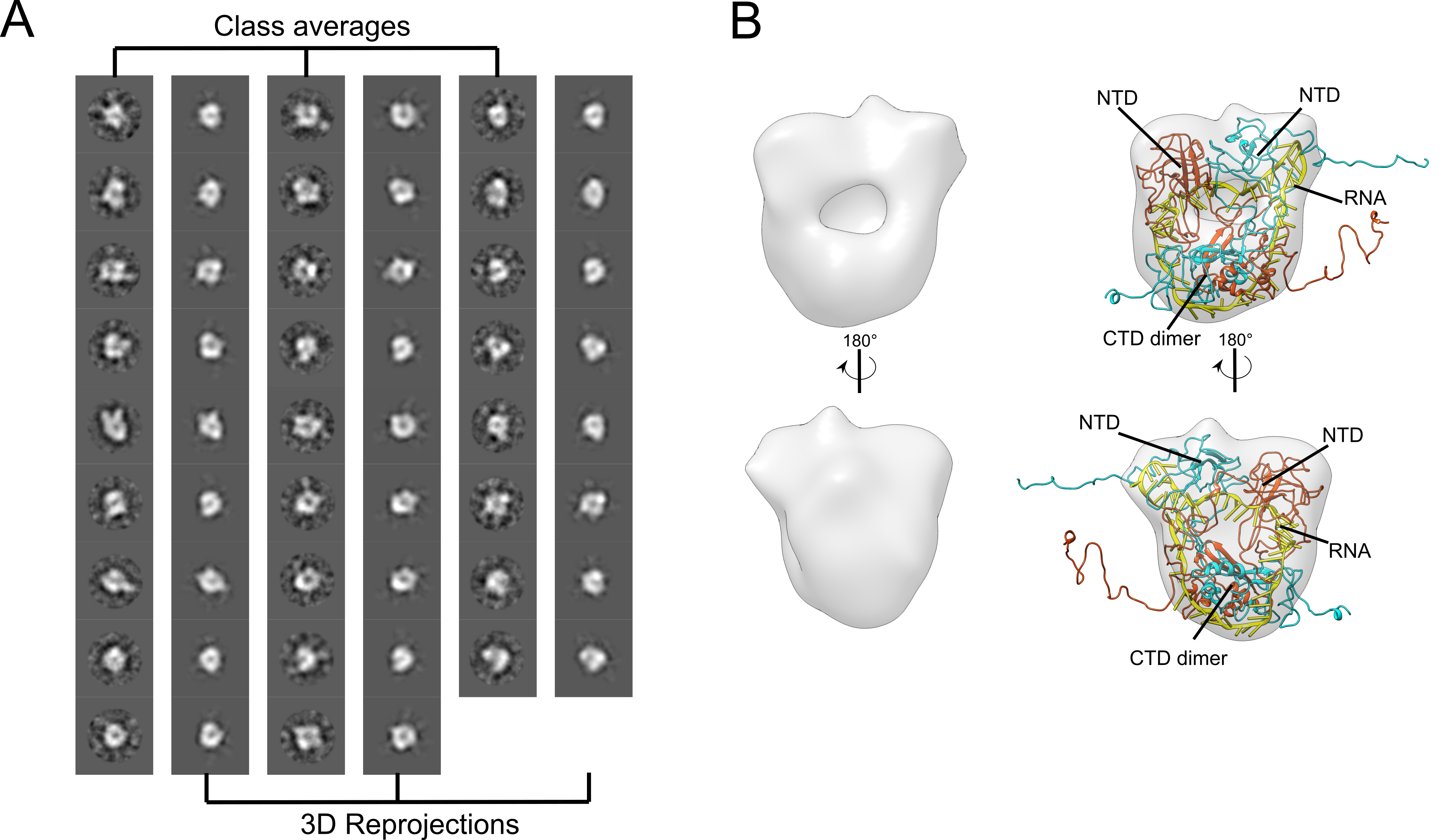

Supplement: S4 Fig — (A) Comparison between toroid-like class averages (even columns) and their reprojections from the 3D volume obtained in EMAN2 (odd columns) to validate the 3D reconstruction. (B) On the left, two different views of the 3D density map reconstruction of the toroid-like particles rendered using 3-sigma contour level. On the right, the same density map with the flexible fitting of the N dimer atomic model derived from the CG simulations performed with the 60 nt-long RNA. All N protein domains are presented. The density map did not present density sufficient for the fitting of N protein CTD C-terminal tails or NTD N-terminal tails, probably due to their high mobility of these domains. One N protein monomer is colored in orange and the other one is colored in cyan. RNA is colored in yellow. (TIF) [file pcbi.1010121.s004.tif]

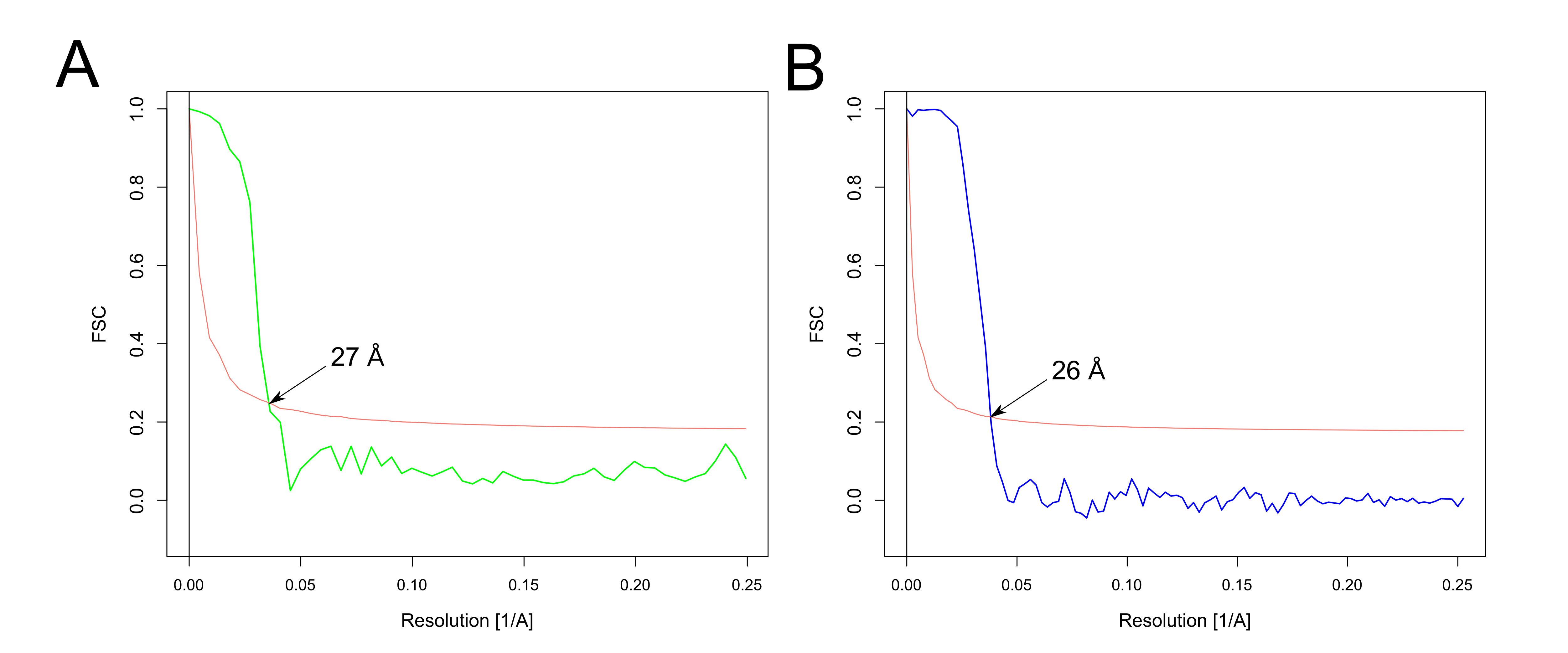

Supplement: S5 Fig — Fourier shell correlation profile of the 3D density maps of (A) toroid-like particles or (B) square-like particles. FSC curve calculated from two 3D volumes is shown in green or blue and the half-bit curve in red. The global resolution determined based on the half-bit criterion is indicated [88]. (TIF) [file pcbi.1010121.s005.tif]

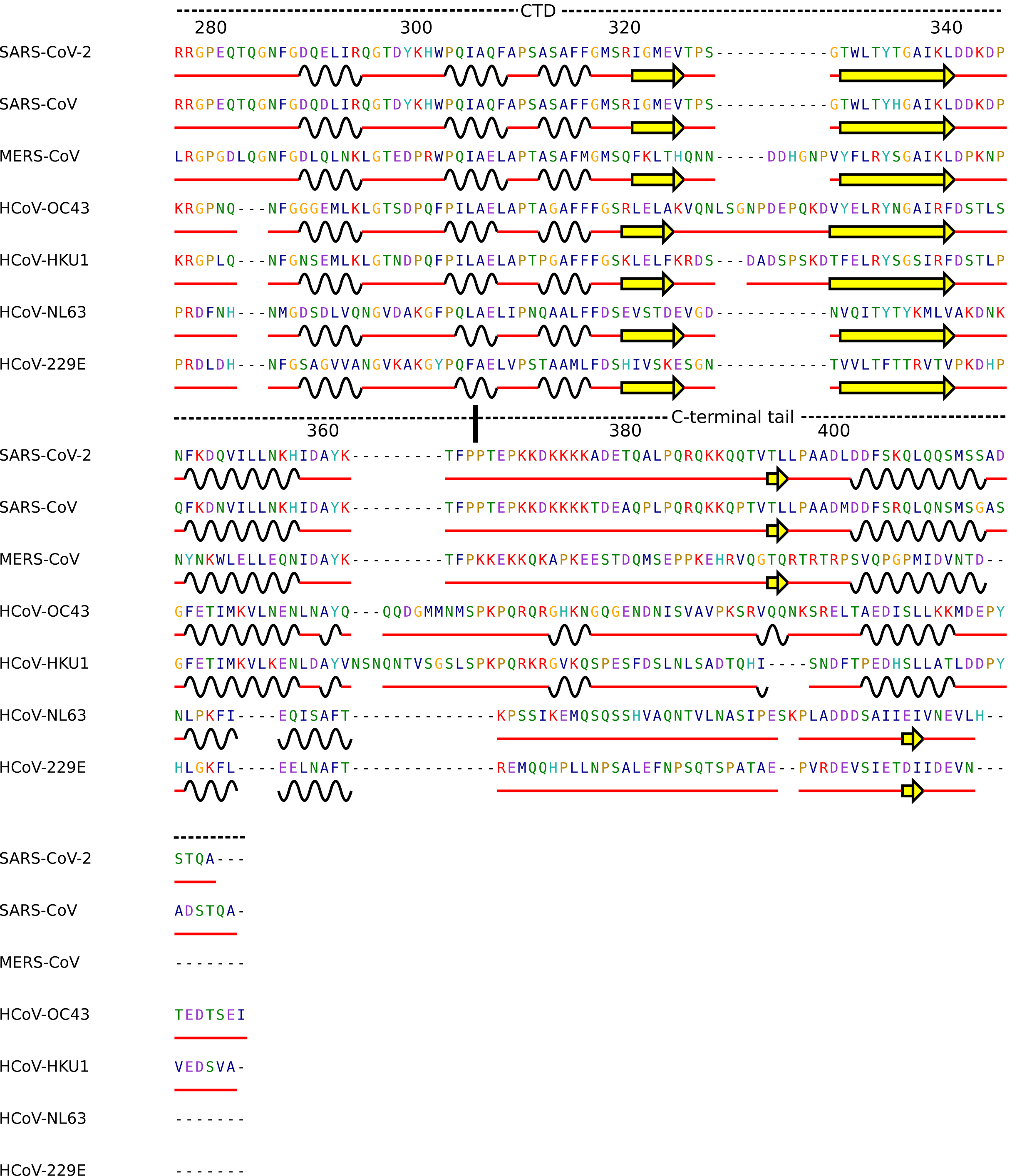

Supplement: S7 Fig — Multiple sequence alignment was performed using Muscle algorithm (3.8) in EMBL-EBI webserver [89]. The alignment was submitted to Ali2D webserver [90,91] and secondary structure prediction for each sequence was generated using PSIPRED algorithm. The multiple sequence alignment and secondary structure prediction was plotted using 2dSS webserver [92]. The numbers indicate SARS-CoV-2 amino acid position and does not consider the gaps. SARS-CoV-2 domains are annotated on the top of alignments. (TIF) [file pcbi.1010121.s007.tif]

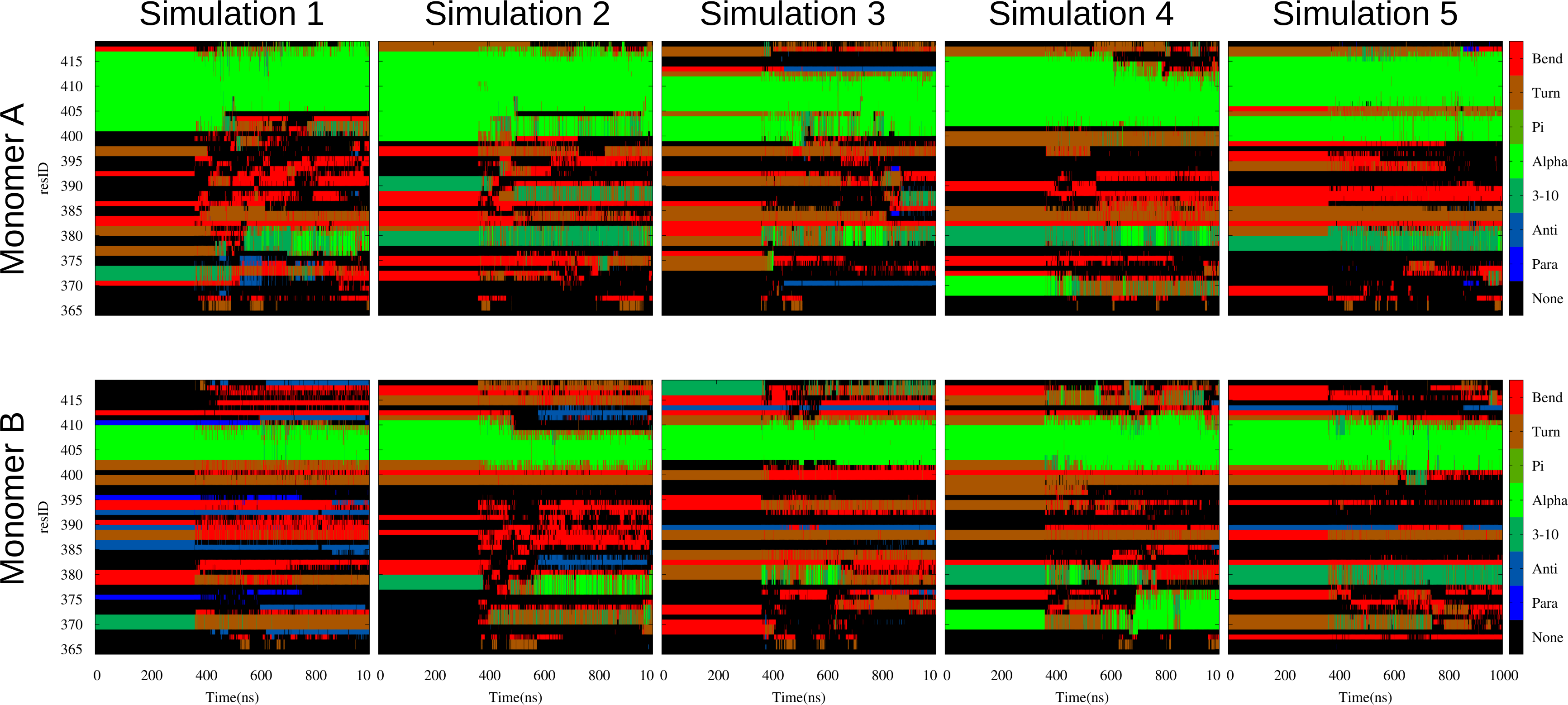

Supplement: S8 Fig — Secondary structure variation by residue for each monomer along simulation time for each of the five replicates and each monomer. The bar shows the color for each secondary structure. The figure was made using secstruct command from CPPTRAJ, a Ambertools20’s program, and GNUPLOT. (TIF) [file pcbi.1010121.s008.tif]

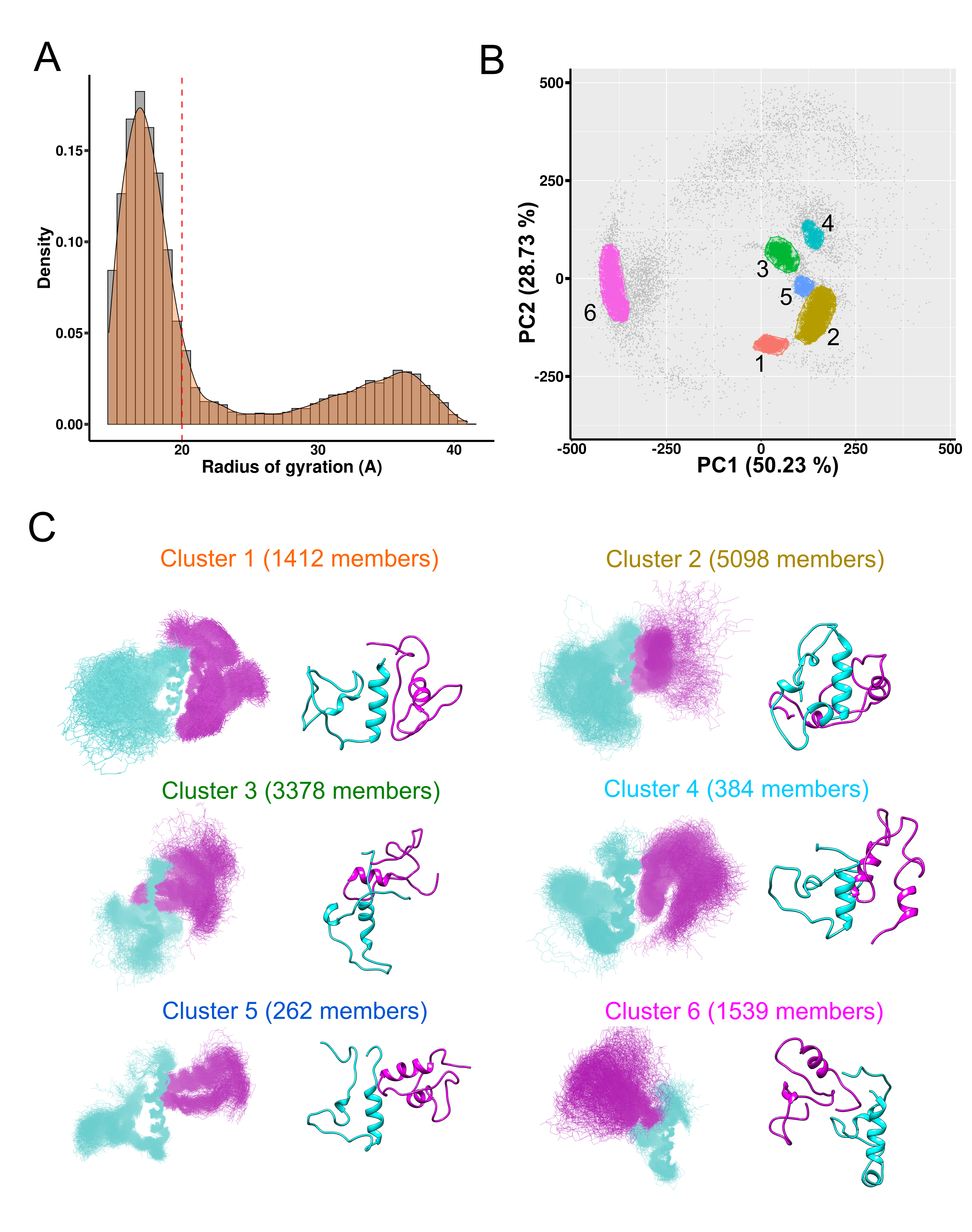

Supplement: S9 Fig — (A) Distribution of radius of gyration of the C-terminal dimeric complex obtained from the entire trajectory. The vertical dashed red line corresponds to the 20 Å cut-off used to select the bound configurations and filter out structures of C-terminal monomers that were not interacting. (B) Principal component analysis obtained from the atomic coordinates of C-terminal bounded configurations. The components PC1 and PC2 were submitted to a clustering analysis with DBSCAN method. The colored points correspond to the six clusters and gray points corresponds to noise. (C) Aligned structures of each ensemble (left) and the representative structure with the lower RMSD in relation to the average structure of the ensemble. (TIF) [file pcbi.1010121.s009.tif]

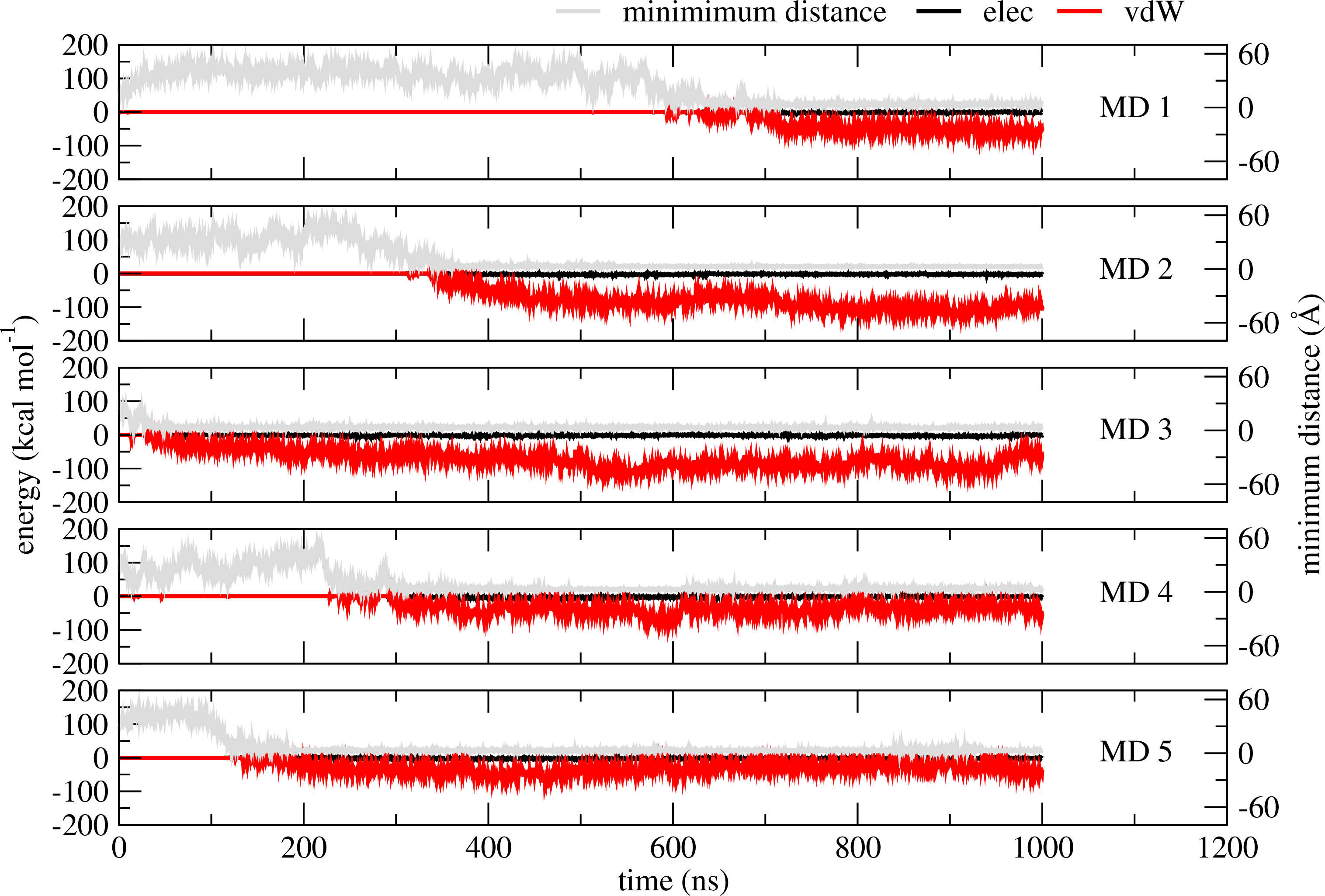

Supplement: S10 Fig — Five independent simulations of the two C-terminal tail monomers in which the dimer is spontaneously formed. The plots show the non-bonded contribution (vdw in red line and electrostatic in black line). The plots suggest the mainly non-bonded contribution is vdw. Minimum distance (in gray line) corresponds to the minimum distance between N protein monomers. (TIF) [file pcbi.1010121.s010.tif]

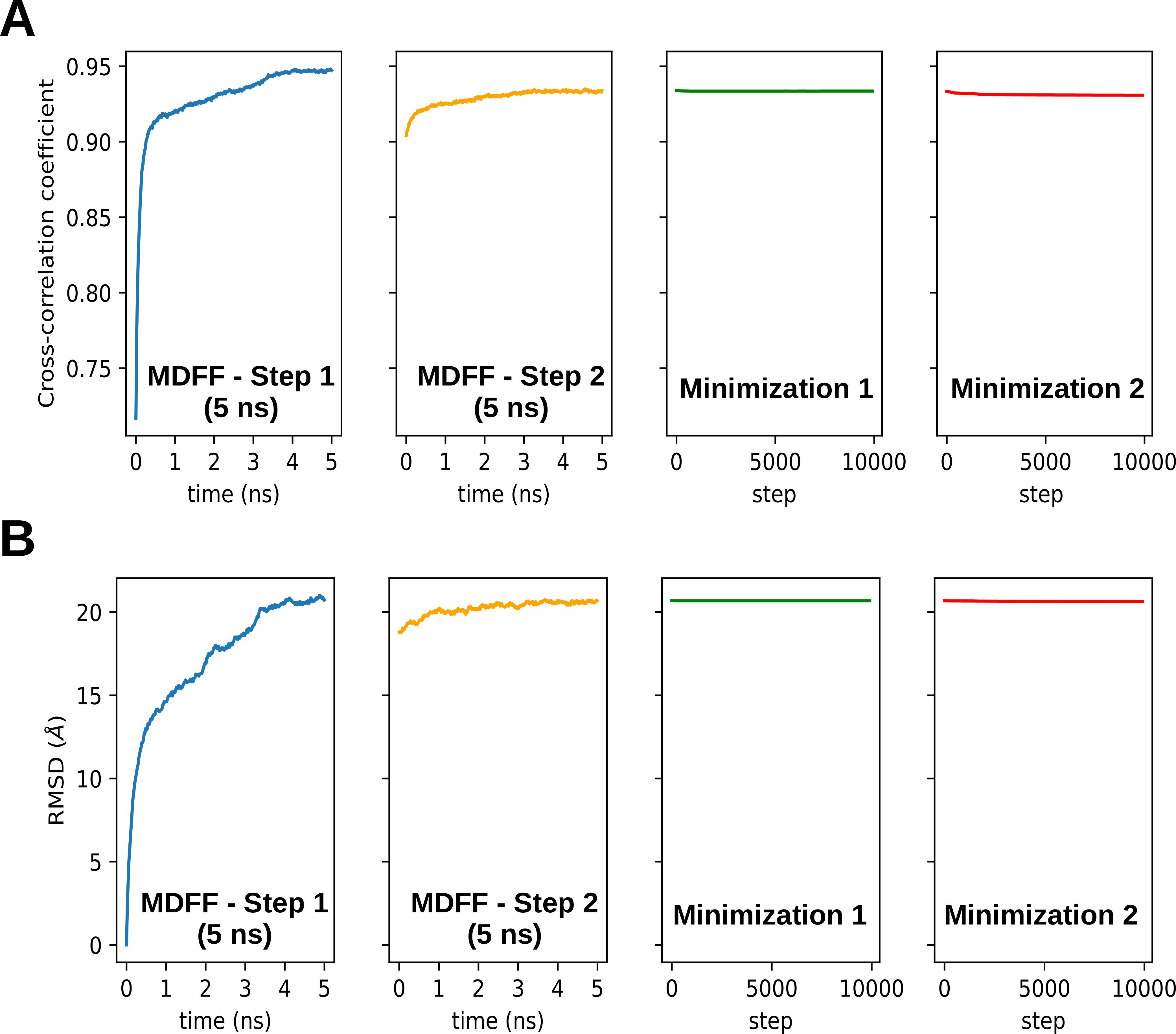

Supplement: S11 Fig — (A) Cross-correlation coefficient (CCC) between model and the 3D density map and (B) RMSD calculations as convergence criteria. Step 1 and Step 2 are MDFF simulations. In Step 1, only the heavy-atom of the RNA and structured domains of the N protein were included in the energy potential of the map, while heavy-atoms of the protein disorder domains were included for Step 2. Minimization 1 is an energy minimization step (10000 steps) with restraints on the protein backbone atom positions, while Minimization 2 is an energy minimization step without any restrain (10000 steps). The CCC and RMSD calculations for Step 1 only include heavy-atoms of RNA and structured domains of the N protein, and for Step 2, Minimization 1, and Minimization 2, heavy-atoms of the RNA and protein. (TIF) [file pcbi.1010121.s011.tif]

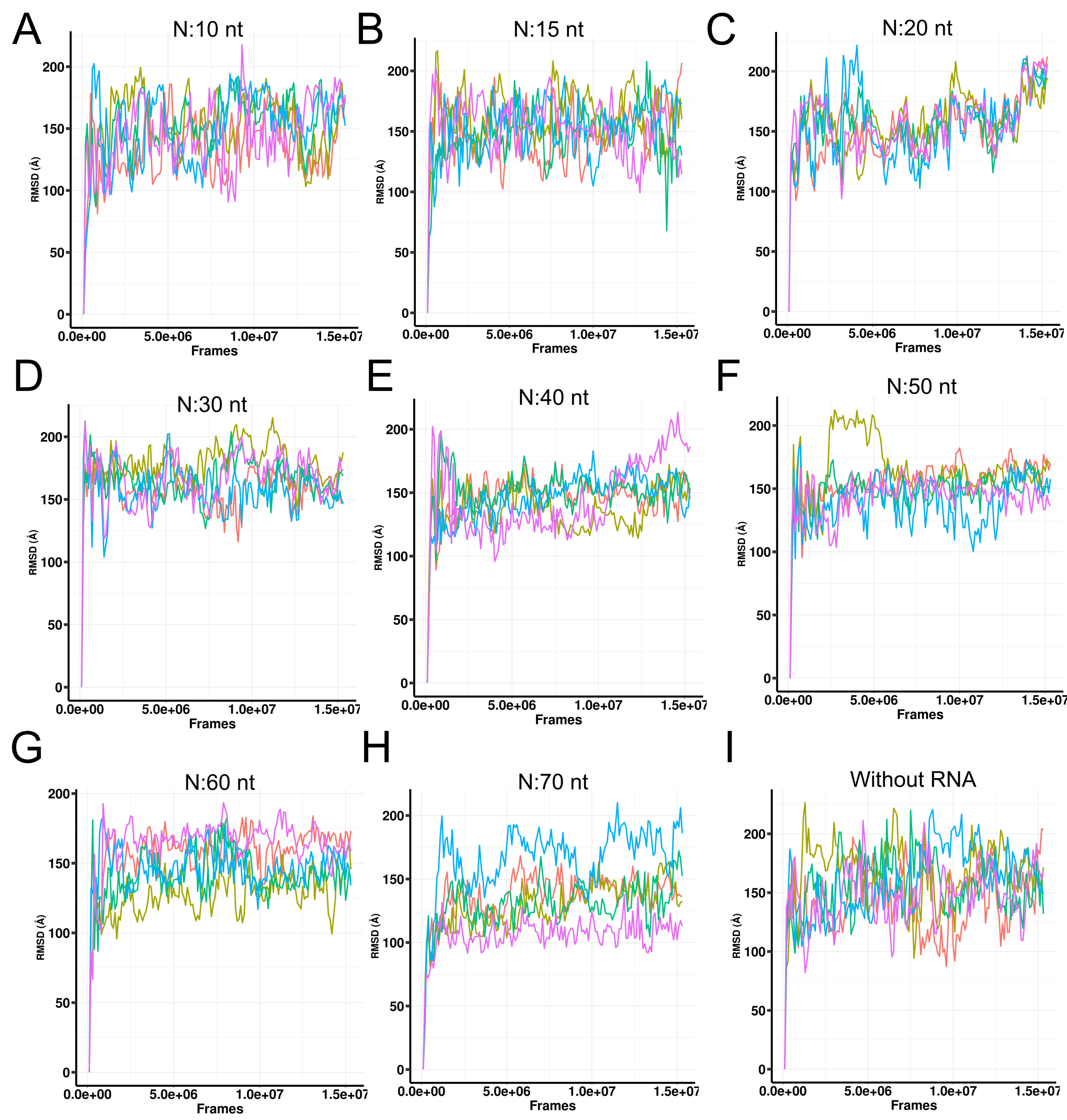

Supplement: S12 Fig — For each condition, the entire trajectory was aligned by the CTD atoms of the N protein dimer using the first simulations frame as reference and the RMSD was calculated using all atoms of the N protein dimer and the first simulations frame as reference. Each line corresponds to a replicate. (TIF) [file pcbi.1010121.s012.tif]

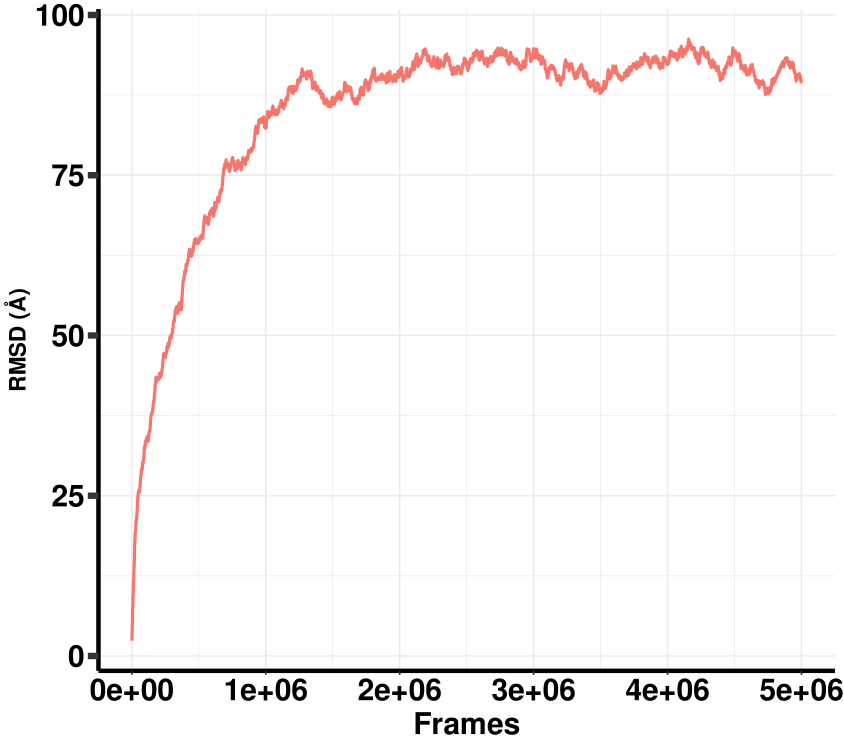

Supplement: S13 Fig — The entire trajectory was aligned by all atoms of the N protein octamer using the first frame as reference and the RMSD calculation was performed using the N protein octamer atoms and the first simulation frame as reference. (TIF) [file pcbi.1010121.s013.tif]

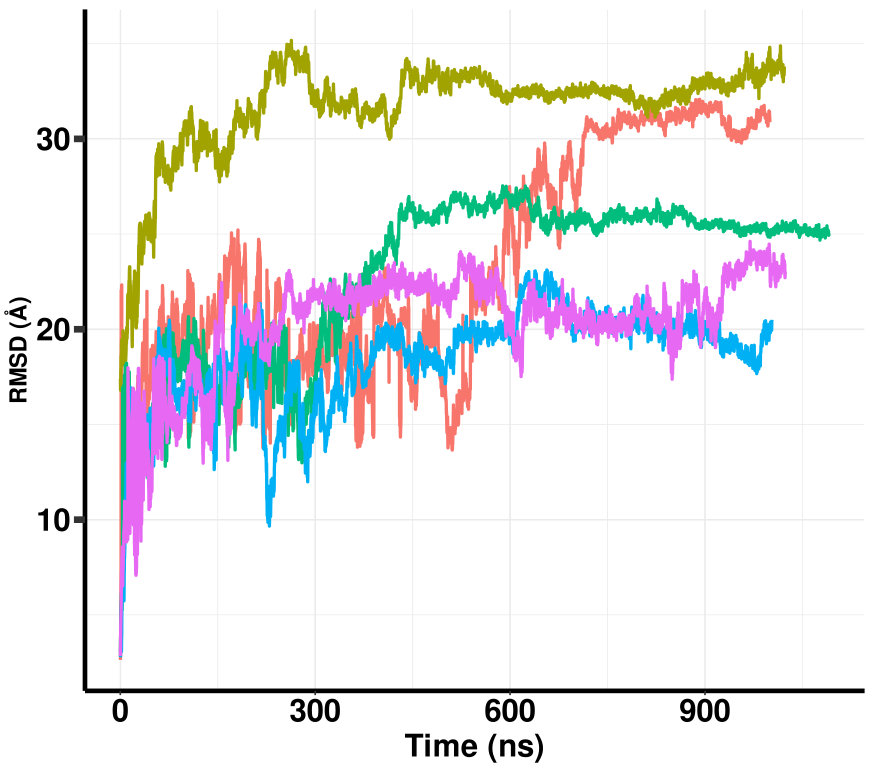

Supplement: S14 Fig — The trajectories were aligned by the backbone atoms of the two CTD-tail monomers the RMSD was calculated using the same backbone selection, and the first frame as reference. Each line corresponds to a replicate. (TIF) [file pcbi.1010121.s014.tif]
