# Supplementary material for: Structural dynamics of SARS-CoV-2 nucleocapsid protein induced by RNA binding
Source: PLoS Comput Biol. 2022 May 12;18(5):e1010121. doi: 10.1371/journal.pcbi.1010121 (PMC9129039; doi:10.1371/journal.pcbi.1010121)
Supplement: Attachment — Submitted filename: PLOSCB_letter_Npro.pdf [file pcbi.1010121.s015.pdf]

LNBio Brazilian Biosciences National Laboratory

CNPEM Brazilian Center for Research in Energy and Materials

18th March 2022

Dear Dr. Alexey Onufriev,

Thank you very much indeed for your response regarding our PCOMPBIOL-D-22 00057 manuscript entitled "Structural dynamics of SARS-CoV-2 nucleocapsid protein induced by RNA binding". We are very grateful for the reviewers' comments and suggestions, which certainly improved the manuscript. Therefore, on behalf of all authors, I'm pleased to submit a point-by-point response letter that outlines remarks raised by the two reviewers with a detailed response to each comment. Changes are highlighted in red in the revised version of the manuscript.

We hope our manuscript is now suitable for publication in PLOS Computational Biology. Please do not hesitate to contact us if you require any further information.

Sincerely,

Dr. Paulo Sérgio Lopes de Oliveira & Dr. Celso Eduardo Benedetti
(paulo.oliveira@lnbio.cnpem.br) (celso.benedetti@lnbio.cnpem.br)

Reviewer's report

Reviewer #1:

In their manuscript, Ribeiro Filho and colleagues describe a thorough study of the coronavirus nucleocapsid protein structure and dynamics. The authors use electron microscopy to get initial insights into the protein organization, and then use modeling to gain understanding at the molecular level. They show that addition of RNA causes compaction of the nucleoprotein particles, in agreement with experimental data, and characterize the protein-RNA complexes containing either a dimer of the N protein or a tetramer of the N protein dimers. Overall, this is a nice and well-written manuscript that presents important new findings about the N protein, which I think should be published after the following minor and technical issues are resolved.

We are very grateful for the reviewer's opinion on our work and we also thank him for the careful evaluation of the manuscript. We made changes to the manuscript according to the reviewer's suggestions, and they are indicated below.

1) Please provide an estimate of the convergence of the simulations reported in Figures 2C, 4, 6B and 7

As suggested by the reviewer, we are providing the estimated convergence of all simulations shown in the Figure 2C, 4, 6B and 7 using the RMSD metric. The resulting plots were included in the new Supplementary Figures S12, S13 and S14. In addition, we have included the following sentences in the Methods section:

Methods section, page 27: "The convergence of CG simulations was accessed by the RMSD calculation. The entire trajectory was aligned by the CTD atoms of the N protein dimer using the first simulations frame as reference and the RMSD was calculated using all the N protein dimer atoms (Figure S12)."

Methods section, page 28: "The convergence of the CG simulation was accessed by the RMSD calculation. The entire trajectory was aligned by all atoms of the N protein octamer using the first frame as reference and the RMSD calculation was performed using the N protein octamer atoms (Figure S13)."

Methods section, page 33: "The convergence of the all-atom simulations was accessed by RMSD calculations. The trajectories were aligned by the backbone atoms of the two

CTD-tail monomers using the first frame as reference and the RMSD calculation was performed using the same atoms selection (Figure S14)."

2) Papers such as this https://doi.org/10.1038/s41467-021-22785-x suggest that significant part of the RNA inside the virion may be double-stranded. Can you provide some discussion and/or opinion whether the N protein also binds double-stranded RNA and how this could affect the structure of RNPs? Does your model agrees with the findings by Caruso et al? (https://doi.org/10.1016/j.bpj.2021.06.003 and https://doi.org/10.1101/2021.07.21.453232)

We thank the reviewer for the questions.

The work of Cao et al. (2021) suggested that the RNA structure in the virion has mainly long-range RNA duplexes. However, the author also indicated that the proposed structural model did not take into account binding information for the N protein. The SARS-CoV-2 genome is densely packaged into the ribonucleoprotein core in the interior of the virion.

Noteworthy, how RNA structures and sequences are recognized by N protein in order to induce a liquid-liquid phase separation and then assembly the nucleocapsid is largely unknown. Some reports suggest that the N protein interacts with ssRNA [Iserman et al. (2020), doi: 10.1016/j.molcel.2020.11.041; Forsythe et al (2022),doi: 10.2139/ssrn.4012175]. On the other hand, other works indicate that the N protein could also interact with structured dsRNA [Roden et al. (2021), doi:10.1101/2021.06.14.448452; Tai et al. (2021) doi: 10.1101/2021.12.25.474155]. Nonetheless, the N protein could likely recognize the dsRNA for later melting it [Caruso et al (2021) doi: 10.1016/j.bpj.2021.06.003; Grossoehme et al. (2009), doi: 10.1016/j.jmb.2009.09.040; McBride et al. (2014) doi: 10.3390/v6082991].

As can be observed in Caruso et al. (2022, doi: 10.1016/j.ijbiomac.2022.01.121) and also reported by Dinesh et al. (2020, doi: 10.1371/journal.ppat.1009100) the binding of dsRNA into the NTD RNA binding site is structurally similar to the binding of ssRNA. In fact, it could be possible that RNPs adopt a different organization when bounded to double-stranded RNAs, but this is still large unknown, especially because the complexity of RNA structural folding, and beyond the scope of our study.

Finally, in our model structural model, the RNA interacts with NTD in a similar way as the presented by Dinesh et al. (2020, doi: 10.1371/journal.ppat.1009100) (Fig. 5) and the interface is also similar to the one reported by Caruso et al. (2022, doi: 10.1016/j.ijbiomac.2022.01.121). For this reason, we decided to cite Caruso et al., as follows:

Results section, page 12: "It is noticeable that, in the proposed model, the interaction of the RNA with the NTD resembles the binding mode of the NTD to a 10-mer RNA reported recently [23] (Figure 5C, lower inset). A similar N protein interaction region formed by the NTD finger-like hairpin was also reported for dsTRS and ssTRS [51]"

3) Have any specific N-RNA interactions been reported? Could they affect the structure of RNPs?

We thank the reviewer for pointing this out. To our knowledge, there is no consensus about specific interaction between the N protein and RNA upon the RNP assembly process. SARS-CoV and SARS-CoV-2 in vitro studies suggested that nucleic acid binds to the N protein in a non-specific and charge-dependent way [Zeng et al. (2020), doi: 10.1016/j.bbrc.2020.04.136; McBride et al. (2014), doi: 10.3390/v6082991]. A recently published NMR structure of SARS-CoV-2 NTD in complex with different RNA sequences (Dinesh et al. 2020, doi: 10.1371/journal.ppat.1009100) indicates that NTD can bind to different RNA lengths and, by analyzing the interface, most of the contacts are through the phosphate group of the RNA and NTD positively charged residues. In fact, several different sequences were demonstrated to bind with high affinity to the N protein [Yang et al. (2021) doi: 10.3389/fchem.2020.624765; Zhao et al. 2021, doi: 10.1016/j.isci.2021.102523]. However, we understand that during the RNP assembly in cells, the preference for some specific sequences in viral genome, such as the package signal sequences, is possibly one of the events that guide the N protein-mediated packaging of the gRNA into RNP the [Lutomski et al. (2021), doi: 10.1021/jacsau.1c00139]. Even so, we believe that non-specific interactions should be necessary to further package the entire viral genome.

Whether specific RNA interactions with N-protein could produce different RNP structure in the context of viral assembly is still to be elucidated. We hope in a future study to address this question.

4) Could it also be that the dimers within an octamer interact through RNA?

We thank the reviewer for this relevant question. Based on our findings and previous studies, our answer to this question is Yes. The N protein oligomerization process, such as octamer formation, seems to be increased in the presence of RNA, especially the longer ones, such as polyA50 (Fig 1). Previous works also corroborate these findings [Zhao et al. 2021, doi: 10.1016/j.isci.2021.102523]. This seems to occur cooperatively with the CTD-tail interaction through dimers, which was shown to be a key event for

protein oligomerization [Chen et al. (2006), doi:10.1021/bi0609319; Ye et al. (2020), doi:10.1002/pro.3909]. Our octameric 3D map indicates that the N protein dimer connects to each other forming a square structure (without contacts between dimers from opposite corners) and the CG model suggests that this is possible through the dimerization of CTD-tails from neighboring dimers. If RNA participates as a bridge or a hub in that interaction is still to be determined.

We included a discussion about this question as following:

Discussion section, page 20: "Likewise, longer RNA segments could drive protein oligomerization, as observed with polyA50 and polyT20 [37]. In this sense, despite the density map of the octameric N protein organization not revealing atomic details, it could be possible that RNA participates in the interactions between dimers within the octamer, in addition to the CTD-tail interaction."

5) Can you please discuss the possible role of N protein interactions with other viral or cellular proteins (such as nsp3 https://doi.org/10.1126/sciadv.abm4034 or 14-3-3 https://doi.org/10.1016/j.jmb.2021.166875)? Could it be that these proteins are also included in the assembled virion and affect the structure of RNPs?

We thank the reviewer for sharing with us these recent publications regarding N protein interaction with other binding protein. The Bessa et al. (2022) results indicate that the N protein may interact with Ubl1 domain from SARS-CoV-2 nsp3 protein through the SR-linker. We think these findings are very relevant specially in the context of viral replication, but to our knowledge there is no clear evidence of its role in the context of viral RNA packaging, which is one of the focuses of our study. Nevertheless, we believe that is important to include a sentence in the discussion section to highlight that the contacts observed between the N protein and RNA can be affected, as demonstrated in the Bessa et al. (2022) study, by the interaction with other protein partners in the viral life cycle, as following:

Discussion section, page 20: "It is important to note that in the context of infected cells, the RNA binding profile could be affected by N protein binding proteins, such as nsp3, as evidenced by [56]."

Additionally, the SAXS results of the abovementioned article is in line with our concept regarding N protein compaction. Thus, we also decided to cite this article, as shown below:

Discussion section, page 19: "According to these models, in the absence of RNA, the N protein oligomerizes into dimers where the NTDs move freely relative to the CTD dimer, as previously suggested [36, 56]."

Similarly, Tugaeva et al. (2021) showed that the human 14-3-3 protein can interact with the N protein particularly when the N protein is phosphorylated. Despite being upregulated in SARS-CoV-2 infection, to our knowledge there is no clear consensus about the exact roles of 14-3-3 in SARS-CoV-2 infection, especially in the context of RNP formation. For this reason, we prefer not to mention this interaction in the discussion section as there is no robust data on the influence of this host cell protein on the viral packaging process.

Finally, to our knowledge there are no reports indicating that non-structural proteins, such as nsp3, or host proteins, such as 14-3-3, which interact with N protein in infected cells could be recruited to be part of the RNP assembly.

page 11, sentence "By fitting the atomic model derived from the CG simulations performed with the 60 nt-long RNA into the 27 Å resolution density map (Figure S5A)" refers to incorrect Figure, should be Figure S4B?

We thank the reviewer for this comment. Actually, our aim was to reference the fourier-shell correlation plot that allowed us to determine the resolution. We changed the sentence to clarify the figure reference, as follows:

Results section, page 12: "fitted into the 27 Å resolution density map (accessed by the fourier-shell correlation in Figure S5A)."

page 12, references to the "right inset": perhaps it woult be nicer to clarify "right top inset", "right bottom inset"

The changes were made according to the reviewers' suggestions:

Results section, page 13-14: "The 2D class average analysis also revealed particles showing a gap between two adjacent rounded units, like a U-shaped particle (Figure 6A, right top inset). Of notice, several square-like particles showed a blurred appendix of similar dimensions as the toroid-like particles (Figure 6A, right bottom inset). Other well-defined patterns comprise particles that seem to be composed of only three rounded units (Figure 6A, left top inset)."

page 13, "This finding was crucial because suggested that square and rod-like particles correspond to different orientations": it is also possible that rod-like particles are simply dimers (of dimers) and not tetramers (of dimers) viewed along their plane

We thank the reviewer for the opportunity to clarify this issue. In our experiments using SEC-MALS we did not observe the presence of N protein tetramers, only dimers and octamers. Moreover, as mentioned in Results section (page 14) and shown in the supplementary figure S6, the 2D reprojections of the 3D density map reconstructed from square-like particles match appropriately the projections of the raw particles (square or rod-like particles) at the same orientation angle. Additionally, in this figure S6, we observe long rod-like particles (180-190 Å) that would be incompatible with dimer of dimers viewed along their plane (that would have ~130 Å along their plane), but are compatible with the diagonal of square-like particles. Thus, we are confident that the rod-like particles correspond to a side-view of octamers.

To clarify this point, we added the followed sentence to the manuscript:

Results section, page 14-15: "Another possibility would be that rod-like particles correspond to dimer of dimers (tetramers) viewed along their plane. However. the size of the longer rod-like particles (ranging from 180 to 190 Å) would not be compatible with the size of dimer of dimers viewed along their plane (~130 Å)."

page 13, "3D density map for the square-like particles (Figure 5C and Figure S5)" probably should be "Figure 6C and Figure S5"

We apologize for this mistake; we corrected the text accordingly.

Reviewer #2:

I have three minor questions/concerns:

We thank the reviewer for having carefully revised the manuscript and for the excellent suggestions. We hope we have satisfactorily addressed all the questions and that now the manuscript is suitable for publication.

1) has the procedure for fitting the CG model been tested elsewhere? If so, it should be cited and discussed. If no, it should be tested if possible on another system for accuracy and minimally discussed as a new method.

We thank the reviewer for the opportunity for pointing this out. The model-to-map fitting process was performed using all-atom structures obtained from the conversion of the CG models to all-atom models. The fitting of all-atom structures into density maps using Situs software is well documented [Wriggers (2012), doi:

10.1107/S0907444911049791]. Thus, we used the conformational sampling of the CG models, which is commonly applied [Tokuhisa et al. (2020), doi:10.1021/acs.jcim.0c00131; Takada (2012), doi: 10.1016/j.sbi.2012.01.010; Yang et al. (2010), doi:10.1073/pnas.1004569107], just to produce initial structures for the fitting. In this way, our sampling and fitting protocol is based on that described for EM-targeted PaCS-Fit [Peng & Zhang (2016), doi: https://doi.org/10.1038/srep29360]. In addition, a similar implementation was successfully applied to interpret low-resolution structural data by combining CG conformational sampling and SAXS data (Yang et al. (2010), doi:10.1073/pnas.1004569107). Moreover, the use of CG models prior to all-atom fitting refinement is also reported in (Kulik et al. (2021), doi: 10.3389/fmolb.2021.631854).

To make the explanation of the process clearer we improved the following sentence:

Results section, page 12: "To better correlate the CG simulations with the negative stain images, we built a low-resolution 3D density map from the toroid-like particles (Figure 5). The comparison between the class averages used to build the 3D map and reprojections generated from the map is presented in Figure S4A. Next, we leveraged the conformational sampling obtained from the CG simulations [47] performed with the 60 nt-long RNA and converted the CG models to all-atom models to select the structures that best fitted into the 27 Å resolution density map (accessed by the fourier-shell correlation in Figure S5A). This process of using a conformational sampling from CG simulations to correlate with experimental structural data was previously applied in [48, 49]. We found that the NTDs are oriented side-by-side facing the CTD dimer (Figure 5B)."

2) from your work, you should be able to get information on the conformational ensemble. Especially if you combined this work with non-parametric clustering. Is there a reason this wasn't done?

We thank the reviewer for this excellent question/suggestion. We accepted the reviewer's suggestion to show information on the conformational ensemble. We performed a principal component analysis (PCA) of the aligned all-atom trajectory of the CTD-tail (Fig. 7) using the Bio3D R package. Then, we created clusters based on the two first principal components using a density-based clustering approach (DBSCAN). The clustering analysis result was really very informative, especially highlighting the pattern of interaction between the two CTD-monomers involving helices. We included the clustering plots and structures presenting the conformation ensemble in Supplementary Figure S10. We also included those results in the Result section, as follows:

Results section, page 16: "Conformational ensembles were obtained by clustering the simulation trajectory into six groups (covering ~70 % of the analyzed frames). The

ensembles evidenced that the C-terminal tails complexes were well-packaged with a stable dimeric interface surrounded by flexible regions (Figure S9). The most frequent interface contacts involved residues 394 to 398 from one monomer with 405 to 412 from the other (Figure 7C and 7F). The residues 394 to 398 correspond to a hydrophobic sequence LLPAA and an energy contribution analysis suggested that van der Waals interactions are the major forces driving the C-terminal tail dimerization (Figure S10). The conformational ensembles, including the most populated one (cluster 2), evidenced the participation of both helices α2 in the C-terminal tail interface (Figure S9)."

The method used to obtain the conformational ensemble was detailed in Methods section, as following:

Methods section, page 33: "To generate a conformational ensemble of the quintuplicate CTD-tail trajectory (~25000 frames), we first estimated the radius of gyration using *rgyr* function from Bio3D R package [66] to remove unbounded complexes (Figure S10A). Then, the bounded complexes (17600 structures) were aligned, using the α-helix comprising residues 93 to 104 from one CTD-tail monomer as a reference, and the atomic coordinates were submitted to a principal component analysis (PCA), using the Bio3D R package. The two principal components (PC1 and PC2 in Figure S9B) were submitted to a density-based spatial clustering analysis with DBSCAN method [86] using the dbscan R package [87] with eps = 15 and number of minimum points = 100 (Figure S9B)."

On the other hand, the CG simulations of the N protein dimer without RNA or in complex with different RNA lengths present a very flexible system, as we mentioned in the manuscript. In addition, the frequency of atomic contacts between NTD and CTD is low which contributes to the low stabilization of the complex in a given conformation. This can be observed in the 3D density map, where one sees an empty space between those domains, indicating no direct interaction. Thus, the CG was important for the understanding of the N protein compaction process induced by RNA, mapping of the main contacts with RNA and guiding the model fitting into the 3D density maps. For these reasons, we believe that a conformational ensemble would not be very informative in this case.

3) see comment under data sharing.

We apologize for the inaccessibility to the repository of the shared data. We have now made it public and it includes the in-house script mentioned in the manuscript.
